# Preparation and performance of alumina/epoxy-siloxane composites: A comparative study on thermal- and photo-curing process

**DOI:** 10.1016/j.heliyon.2024.e27580

**Published:** 2024-03-04

**Authors:** Chan Soo Kim, Junho Jang, Hyeon-Gyun Im, Seogyoung Yoon, Dong Jun Kang

**Affiliations:** a Insulation Materials Research Center, Electrical Materials Research Division, Korea Electrotechnology Research Institute (KERI), Changwon-si, 51543, Republic of Korea; b School of Materials Science and Engineering, Pusan National University, Busan, 46241, Republic of Korea

**Keywords:** Epoxy-siloxane composites, Alumina particle, Thermal conductivity, Electronic packaging

## Abstract

Although epoxy-based composites that consist of inorganic fillers and matrixes are widely used in “conventional” electronic packaging applications due to their excellent insulation and robust properties, they limit their uses in “advanced electronic packaging” which requires enhanced thermal conductivity. However, conventional thermal curing methods for fabrication of epoxy-based composites do not fulfill sufficient thermal conductivity. Herein, we apply photo-induced curing strategy for fabricating alumina-incorporated epoxy-siloxane composites that consist of sol-gel derived siloxane matrix and bimodal sized alumina particles as a thermally conductive filler. We investigate how curing mechanism (thermal- or UV-curing) and varying the ratios of the alumina particles of two different sizes affect the various physical properties. It is found that photo-curing process makes greatly enhanced thermal conductivity, low thermal expansion, and high mechanical robustness compared to thermally-cured composites. As the results, we can achieve significantly enhanced thermal conductivity (>11 W/m K) with high thermal stability and mechanical robustness.

## Graphical abstract

**Figure:**
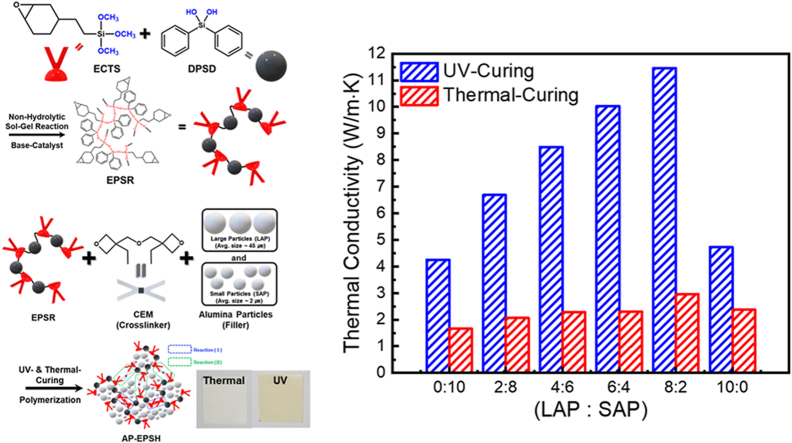

## 1 Introduction

Epoxy-based polymeric materials have been extensively applied to a wide range of fields, for example paints, coatings, adhesives, aerospace industry and electronics materials because of their high mechanical properties, good thermal/chemical resistances, and high adhesiveness to versatile substrates [[1], [2], [3]]. Although they are widely used as electronics packaging materials, the properties of conventional epoxy-based materials (e.g., bisphenol A type diglycidyl ether) do not meet the requirements of advanced electronics materials, which include high thermal conductivity, stability, mechanical properties, and electrical performance [[4], [5], [6]]. A high thermal conductivity is critical for decreasing internal overheating, which can generate cracks or deformations in the microlevel structure of high-efficiency electronics devices [7]. These cracks and deformations can cause system failures, human health and safety problems, and assembly loss in the electronics components [8,9].

Incorporating filler materials with high thermal conductivity into an epoxy-based polymeric matrix to form composites is an effective method to improve thermal performance in cases where the thermal conductivity of epoxy-based materials is too low for removing the heat generated by electronics devices. Typical thermally conductive filler materials include metallic, ceramic particles, and carbon-based materials (e.g., carbon nanotubes and graphene) [7,8,10,11]. Among various thermally conductive fillers, alumina particles (APs) have utilized as filler materials for the thermal management of electronics because of their high thermal conductivity (30 W/m K), and cost effectiveness [10,[12], [13], [14], [15]]. However, the traditional strategy for fabricating AP-incorporated epoxy-based composites limits to enhancing the thermal performance of composites because of the issues of the uniform distribution of AP in matrix. Moreover, conventional epoxy-based polymeric materials show low thermal stability and other physical properties, which hinder uses in advanced electronic devices [5].

Improving thermal conductivity of composites consisting of inorganic fillers and polymer matrices can be achieved by percolation of inorganic fillers; the effective thermal pathway that can be generated by proper contact of inorganic fillers makes enhanced thermal conductivity of the composites. Thus, uniform distribution of inorganic fillers in the composites is crucial for effective percolation; aggregated and/or isolated fillers cannot contribute to the thermal pathway. To attain uniform dispersion of inorganic fillers, optimal fabrication methods can be considered because typical fabrication methods of composites suffer from sedimentation of inorganic fillers which may hinder thermal percolation while curing process of organic matrices. Thus, preventing particle's sedimentation should be guaranteed while fabrication process.

Photo-curing process by ultraviolet (UV) irradiation (UV-curing) is often used for fabricating various composites due to its fast curing time and low shrinkage [16,17]. Photo-cured composites can ensure uniform composite structure compared to thermal cured; inorganic fillers can be uniformly distributed due to UV-induced fast curing of organic matrices, so particle's sedimentation can be prevented. For fabricating electronic packaging composites, however, photo-curing has been rarely utilized because typical epoxy-based polymer matrices in electronic packaging require sufficient thermal energies to be completely cured.

Organic–inorganic siloxane hybrid materials derived from sol-gel hydrolysis and condensation have been utilized widely in the past few decades because of their various advantages including high optical properties and excellent thermo-mechanical characteristics [16,18,19]. Given these high performances, siloxane hybrid materials have been used in a wide range of applications, for example, flexible substrates [20], functional coatings [21], encapsulation of nanocrystals [22,23], dielectrics [24], and electronics packaging [3,5]. In particular, thermal stability of siloxane hybrid materials is considerably higher than that of conventional polymers containing epoxy-based materials because they have robust inorganic siloxane networks (composed of Si–O–Si bonds) that have a higher bond energy than the carbon bond (bond energy: Si–O = 452 kJ/mol, and C–C = 346 kJ/mol) [25]. Furthermore, other physicochemical properties, such as chemical inertness and mechanical durability, can also be obtained with thermal stability. In addition, some organo-siloxane matrices, such as cycloaliphatic epoxy-based siloxane, can be applied to UV-curing process [17].

Based on the above considerations, we developed UV-curable and robust alumina-incorporated cycloaliphatic epoxy-based composites (AP-EPSH) with superior thermal conductivity (>11 W/m K) for electronic packaging. The composites consist of bimodal sized micro-alumina particles (APs) (average size of 2 μm (SAP) and 45 μm (LAP)) with fillers and UV-curable cycloaliphatic-epoxy-siloxane (EPSH) matrix with high thermal stability. For uniform distribution of the AP fillers in the composites, the size and composition of AP fillers are optimized based on the theoretical packing density rule. For robustness and photo-curability of the composites, cycloaliphatic-epoxy-siloxane matrix is utilized owing to its capability of cationic curing. We investigated the changes in thermal, mechanical, and electrical performances by varying size and composition of bimodal sized APs in the siloxane matrix. In addition, different curing mechanisms (UV- and thermal-curing) are implemented for fabrication of the composites to assess the effects on subsequent thermal, mechanical, and electrical properties.

## 2 Materials and methods

### 2.1 Materials

Diphenylsilanediol (DPSD, Gelest, USA), 2-(3,4-epoxycyclohexyl)ethyltrimethoxysilane (ECTS, Gelest, U.S.A.), 3-ethyl-3 [(3-ethyloxetane-3-yl)methoxy]methyl oxetane (DOX, Toagosei, Japan), APs (SAP and LAP) (AnHui Estone Material Technology Co., Ltd., China), triarylsulfonium hexafluoroantimonate salt (Sigma-Aldrich, U.S.A.), 2-methylimidazole (Sigma Aldrich, USA), and barium hydroxide monohydrate (Ba(OH)_2_·H_2_O, Sigma Aldrich, USA) were used without any further purification.

### 2.2 Synthesis of cycloaliphatic epoxy-phenyl-based siloxane hybrid resin (EPSR)

A Cycloaliphatic epoxy-phenyl siloxane matrix resin (EPSR) was synthesized by a nonhydrolytic sol–gel condensation reaction following previously reported methods [5,16]. ECTS and DPSD (molar ratio of 1:1) were blended in a two-neck flask using a magnetic (or mechanical) stirrer for a few minutes. Next, 0.2 mol% Ba(OH)_2_·H_2_O of total silane precursors was added to promote a base-catalyzed sol–gel reaction between the ECTS and DPSD. The blended solution was mixed at 80 °C under nitrogen purging for a few hours. After mixing, a viscous siloxane resin was obtained.

### 2.3 Fabrication of alumina-incorporated epoxy-based siloxane composites (AP-EPSH)

The DOX as an epoxy crosslinker was added to EPSR at a 2:3 M ratio of the epoxy molecule. Triarylsulfonium hexafluoroantimonate salt (for UV-curing) or 2-methylimidazole (for thermal-curing) was added to induce the epoxy ring-opening polymerization at 1 wt% of total resin content. Further, APs (SAP and LAP) were added to the mixed resin at a certain concentration (90% of matrix) (the ratio of SAP to LAP is varied from 0:10 to 10:0). The final mixed resin was exposed to UV-lamp (240 mJ/s, 10 min) for UV-curing or was stored at 120 °C for 5 h for thermal-curing to obtain the cured product (UV- or thermal-cured AP-EPSH) was obtained.

### 2.4 Characterization

Thermal properties were examined by thermogravimetric analysis (TGA, Q50, TA Instruments, Inc.) at a heating rate of 10 °C/min under N_2_ atmosphere. Thermomechanical properties were measured by thermomechanical analysis (TMA, EXTAR TMA/SS 6100, Seiko Instrument, Inc.) at a heating rate of 10 °C/min. Fourier-transform infrared (FT-IR) spectra were recorded with on an FT-IR 680 plus instrument (JASCO, U.S.A.). ^29^Si nuclear magnetic resonance (NMR) spectra were obtained using a DMX600 FT 600-MHz spectrometer (Bruker Biospin, Australia). Mechanical properties were measured using a universal testing machine (AGS-X STD, Shimadzu, Japan). The dielectric properties of the composites (at 5 Hz) were measured using a microdielectrometer (AET, Japan). The surface resistivity of the composites was obtained using an electrometer (6517 B, Keithley, USA) and a resistivity test fixture (8009, Keithley, USA). The breakdown strengths of the composites were recorded using a breakdown strength tester (SKY-HAV-1010, SKY Innotek, Republic of Korea). The thermal conductivity was determined using a thermal conductivity analyzer (LAF 6720, C-Therm Technologies, Canada). The SEM images were obtained using scanning electron microscopy (S-4800, Hitachi, Japan)

## 3 Results and discussion

### 3.1 Fabrication of AP-incorporated epoxy-based siloxane composites

**Fig. 1:**
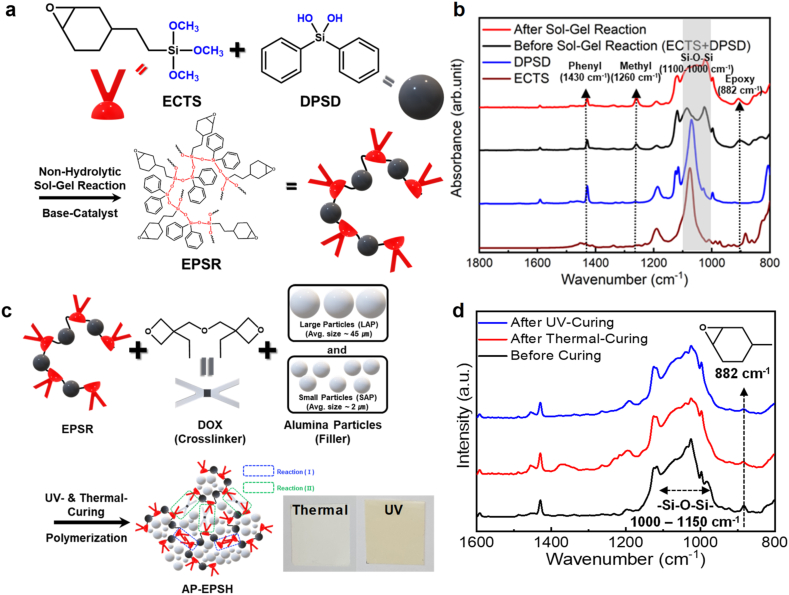
a) Synthesis scheme of EPSR and b) comparison of FT-IR spectra before (ECTS, DPSD and mixture) and after sol-gel reaction. c) Fabrication scheme of AP-EPSH and d) comparison of FT-IR spectra before and after thermal curing.

Fig. 1a presents the procedure for synthesizing cycloaliphatic epoxy-phenyl-based siloxane hybrid resin (EPSR) via a non-hydrolytic sol–gel reaction between the methoxy group of ECTS and the hydroxyl group of DPSD. Unlike a conventional hydrolytic sol–gel reaction, non-hydrolytic sol–gel reactions do not require water for hydrolysis of silane precursors prior to condensation; however, siloxane networks can be formed because of the existence of the hydroxyl group (silanol) in DPSD [26]. The FT-IR spectra (Fig. 1b) shows that the band corresponding to the stretching of the siloxane bond (Si–O–Si) at 1100–1100 cm^−1^ is considerably broadened after the non-hydrolytic sol–gel reaction, indicating the successful formation of siloxane networks, which can be further confirmed by comparing FT-IR spectra of ECTS, DPSD and EPSR. In addition, the organic groups present in the two types of silane precursors (epoxy group peak at 882 cm^−1^; and phenyl group peak at 1430 cm^−1^) remained after the sol–gel reaction, indicating that they did not decompose during the non-hydrolytic sol–gel reaction. In the sol–gel reaction between ECTS and DPSD, there are two reactive functional groups to form siloxane networks in which three methoxy groups and two hydroxyl groups present in ECTS and DPSD, respectively, thereby randomly formed siloxane bonds with branched structure can be generated. These can be confirmed in FT-IR spectra, where the absorbance intensity at the peak region in 1000–1100 cm^−1^ is much broaden and no distinct peaks in the siloxane bond region. Moreover, synthesized EPSR showed a high degree of siloxane bond formation (∼85%) that can be observed in the ^29^Si-NMR results, supporting the random and branched siloxane bond formation (Fig. S1).

Using EPSR, we fabricated AP-incorporated siloxane hybrid-based composites (AP-EPSH) by combining LAPs and SAPs (Fig. 1c) having average sizes of 2 and 45 μm, respectively (Fig. S2). The weight ratio of AP to EPSH is 90:10. Keeping the total weight concentration of AP constant, we changed the ratio of LAP to SAP (from 0:10 to 10:0). For complete epoxy curing, the di-oxetane monomer (DOX) that acts as an epoxy crosslinker as well as a hardener, was used as the curing agent. Subsequently, after mixing crosslinker (DOX) and bimodal sized APs (LAP and SAP) with the matrix, AP-EPSH was fabricated via thermal- or UV-induced cationic epoxy ring-opening polymerization [5,16]. In this process, there are two chemical crosslinking reaction: (Ⅰ) a chemical reaction between epoxy-functional groups in siloxane matrix and (Ⅱ) a chemical reaction between epoxy-functional group in siloxane matrix and oxetane group in DOX. These two chemical crosslinking reactions induce the formation of alumina/epoxy-based siloxane composites with rigid and high crosslinking density [5,22,25]. Fig. 1d presents the FT-IR spectra before and after the thermal- and UV-induced epoxy curing process; the peaks correspond to the epoxy ring at 882 cm^−1^ was removed completely, indicating that APs did not interrupt the epoxy ring-opening process. Therefore, we fabricated successfully cured AP (with two different size)-incorporated cycloaliphatic epoxy-phenyl-based siloxane composites.

### 3.2 Morphology and packing density of AP-EPSH

**Fig. 2:**
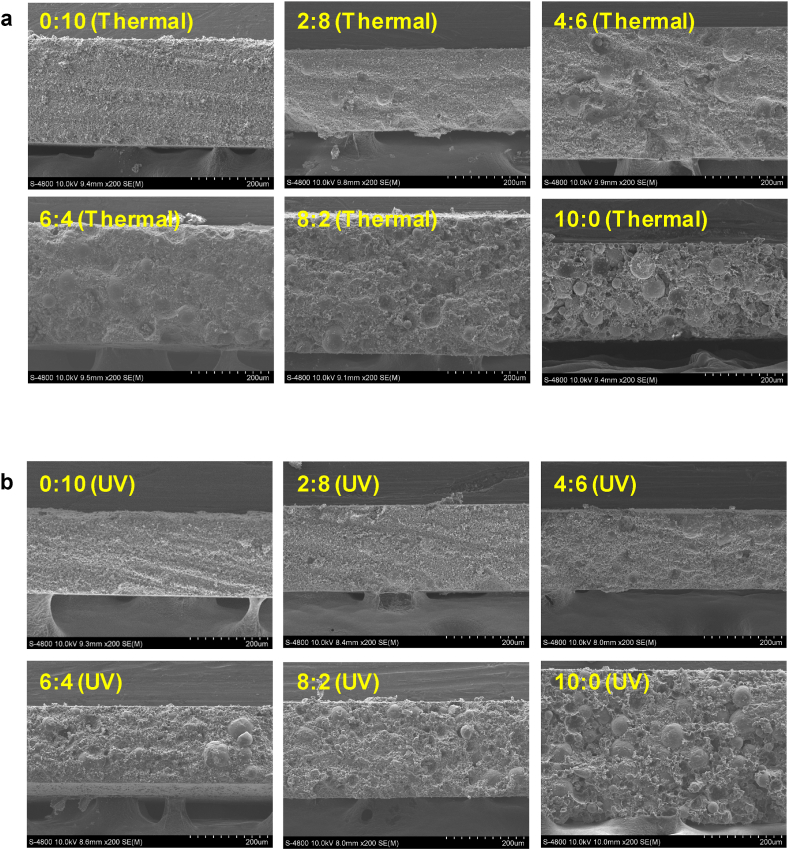
(Revised). Cross-sectional SEM images of a) thermal- and b) UV-cured AP-EPSH according to changing the ratio of LAP to SAP.

The photographs of AP-EPSH for different ratios of SAP to LAP are shown in Fig. S3, which shows the increased optical haze causes by APs incorporation. Fig. 2 shows cross-sectional SEM images of thermal-cured (Fig. 2a) and UV-cured (Fig. 2b) AP-EPSH by varying the ratio of LAP to SAP, in which all samples according to two different curing mechanism and varying the ratio of LAP to SAP exhibited distribution of APs surrounded by the EPSH matrix was confirmed. Especially, UV-cured composites showed more homogeneous distribution of APs in EPSH matrix than that of thermal-cured samples.

Before the investigation of various physical properties, we confirmed the packing density of composites with two different sized filler particles. In conventional particle packing theories, loosening (large particle dominant case) and wall (small particle dominant case) effects are considered to obtain the theoretical calculation values of the packing density of power, which exhibit highly reliable prediction [[27], [28], [29], [30], [31], [32]]. The packing density of powder after mixture can be expressed by

(1) $$\rho =\sum_{i=1}^{n}\emptyset_{i}\;\left(i=1\;\text{for}\;\text{small}\;\text{particle},\text{and}\;2\;\text{for}\;\text{large}\;\text{particle}\right)$$

where *ρ* and *φ*_*i*_ represent the packing density using two different size particles and partial volume fraction of given particles (small or large particles), respectively. The fractions of large or small particles are denoted by

(2) $$\omega_{i}=\frac{\emptyset_{i}}{\rho }\text{,}$$

(3) $$\sum_{i=1}^{n}\omega_{i}=1\text{.}$$

When small particles in the matrix are dominant, the space occupied by large particles can be defined as *φ*_*2*_; therefore, the space occupied by small particles is 1 − *φ*_*2*_. If there are no interactions between large and small particles, *ρ* is expressed by

(4) $$\rho =\emptyset_{2}+\alpha \left(1-\emptyset_{2}\right)\text{,}$$

where α represents the packing density of both particles. When small particles in the matrix are dominant, the *ρ* of power (denoted by *ρ*_*1*_) considering the wall effect is obtained by [29]:

(5) $$\rho_{1}=\alpha_{1}+\left(1-\alpha_{1}\right)g\emptyset_{2}\text{,}$$

where *g* represents the wall effect related to the constant determined by *g* = 1 − *r*_*1*_*/r*_*2*_, and *α*_*1*_ represents the packing density of small particles [29]. When *r*_*1*_*/r*_*2*_ is closed to zero, small particles are too small to consider the wall effect. To make Eqs. (4), (5) consistent, *g* equals to 1. When the size of large particles is the same with that of small particles (i.e., *r*_*1*_*/r*_*2*_ = 1), *ρ*_*1*_ is equal to *α*_*1*_ because only small particles are considered, the *g* value is zero. When large particles in the matrix are dominant, *ρ* without the interaction is expressed as

(6) $$\rho =\alpha_{2}+\emptyset_{1}$$

where *α*_*2*_ represent the packing density of large particles. Considering the loosening effect, the *ρ* of binary particle system (denoted by *ρ*_*2*_) can be obtained from [29]:

(7) $$\rho_{2}=\alpha_{2}+f\emptyset_{1}\text{,}$$

where *f* represents the loosening effect-related constant. Parameter *f* is similar to *g* in which *f* = 1 when *r*_*1*_*/r*_*2*_ = 0 and *f* = 0 when *r*_*1*_*/r*_*2*_ = 1.

**Fig. 3:**
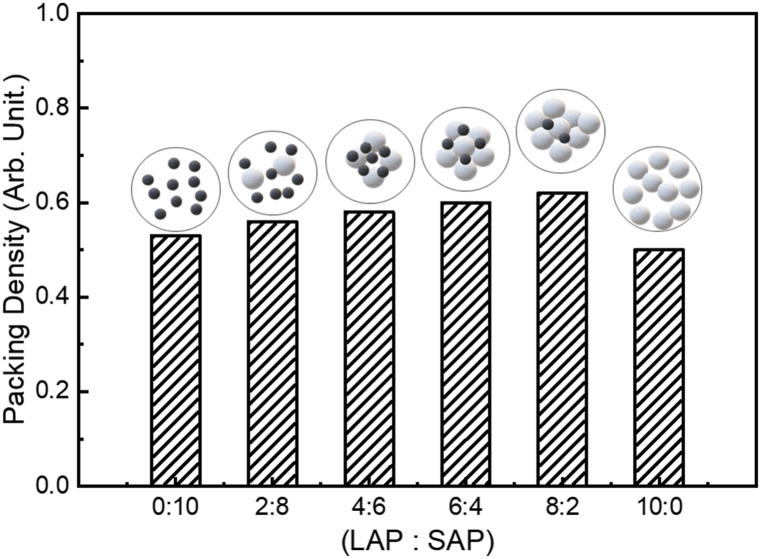
Changes of packing density of AP-EPSH according to the ratios of LAP to SAP.

Based on packing theories, we calculated the packing density using the various physical parameters of LAP and SAP (Fig. 3). We used specification values from manufacturers and calculated the volumes of two different APs using their average diameters. Therefore, the highest packing density of AP-EPSH was obtained when the ratio of LAP to SAP was 8:2.

### 3.3 Thermal properties of AP-EPSH

**Fig. 4:**
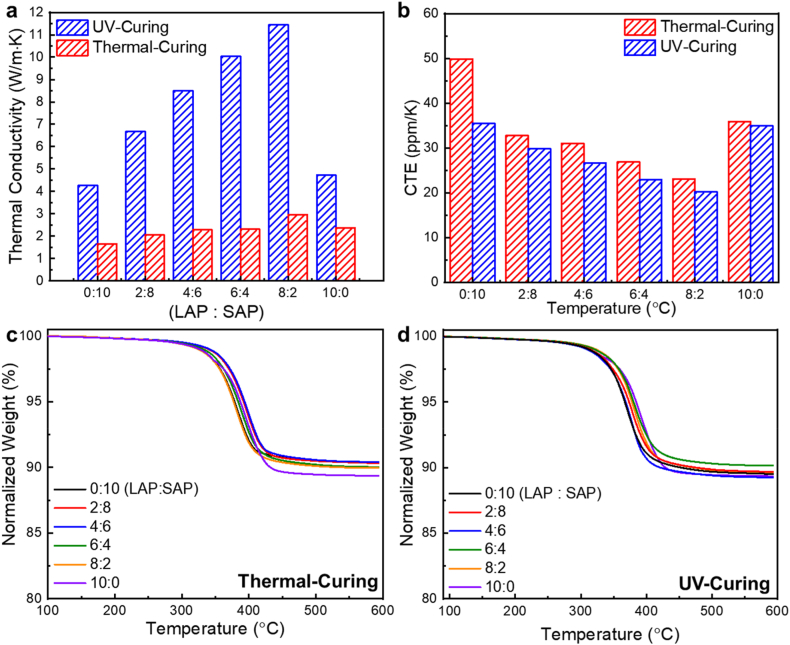
Thermal properties of AP-EPSH according to the ratios of LAP to SAP. a) thermal conductivity, b) coefficient of thermal expansion, TGA results of AP-EPSH using c) thermal-curing and d) UV-curing.

We investigated changes in the thermal conductivity of AP-EPSH by thermal- and UV-curing processes for different ratios of LAP to SAP following the incorporation of AP with a high-thermal conductivity filler (Fig. 4a). By increasing the LAP concentration until 80% (LAP:SAP = 8:2), the thermal conductivity of UV-cured AP-EPSH was increased dramatically (from 4.26 to 11.45 W/m K) that is much higher than those of thermal-cured composites (from 1.66 to 2.97 W/m K). This is because UV-cured composite retains uniform distribution of LAPs and SAPs compared to thermal-cured sample, which induces effective thermal conductive pathways making high thermal conductivity. Fig. S4 represents schematic illustration of difference of thermal conduction of between thermal- and UV-cured composites. On the other hands, thermal-cured composites have large portion of sedimented particle in matrix because the thermal-curing process can cause aggregation and/or agglomeration of particles, which inhibits thermal conduction that cause low thermal conductivity. This can be also confirmed by previous cross-sectional SEM images of UV- or thermal-cured AP-EPSH (Fig. 2). The thermal conductivity values of all AP-EPSH were higher than that of the previous reported epoxy-based siloxane/silica composites because of the higher thermal conductivity of AP than that of silica (AP = 30 W/m K, silica = 12–20 W/m K) [5]. When LAPs were further added in AP-EPSH (i.e., LAP:SAP = 10:0), the thermal conductivity values of UV- and thermal-cured AP-EPSH were reduced due to decreasing packing density. Although the decreasing thermal conductivity of composites by further added LAPs, UV-cured AP-EPSH still exhibited higher than thermal-cured EPSH.

We compared the thermomechanical properties of UV- and thermal-cured AP-EPSH by varying the ratio of LAP to SAP (Fig. 4b and S5). The coefficient of thermal expansion (CTE) values of AP-EPSH gradually decreased on increasing the LAP portion until the ratio of LAP to SAP is 8:2 because of the increasing packing density. The CTE values of UV- and thermal-cured AP-EPSH were increasing when LAP was added to 100%, caused by decreasing the packing density. All CTE values of UV-cured AP-EPSH according to varying the ratio of LAP to SAP were higher than those of thermal-cured samples because UV-curing process induces uniform distribution of APs making effective preventing thermal expansion of composites. We investigated thermal stability by confirming thermal decomposition behavior using TGA, which exhibited high five weight percent loss temperature (T_d5wt%_) (∼380 °C) and residual weight (∼90%) that is higher than those of bare siloxane matrix and commercial epoxy-based matrix, which are attributed to robust siloxane matrix and highly concentrated inorganic fillers [5] (Fig. 4c and d and Table S1). Therefore, we attributed that incorporating APs induces greatly enhanced thermal stability and the packing density affected the thermal expansion property. In addition, UV-cured composites exhibited much higher thermal conductivity (>11 W/m K) than that of thermal-cured AP-EPSH (∼3 W/m K).

### 3.4 Mechanical properties of AP-EPSH

**Fig. 5:**
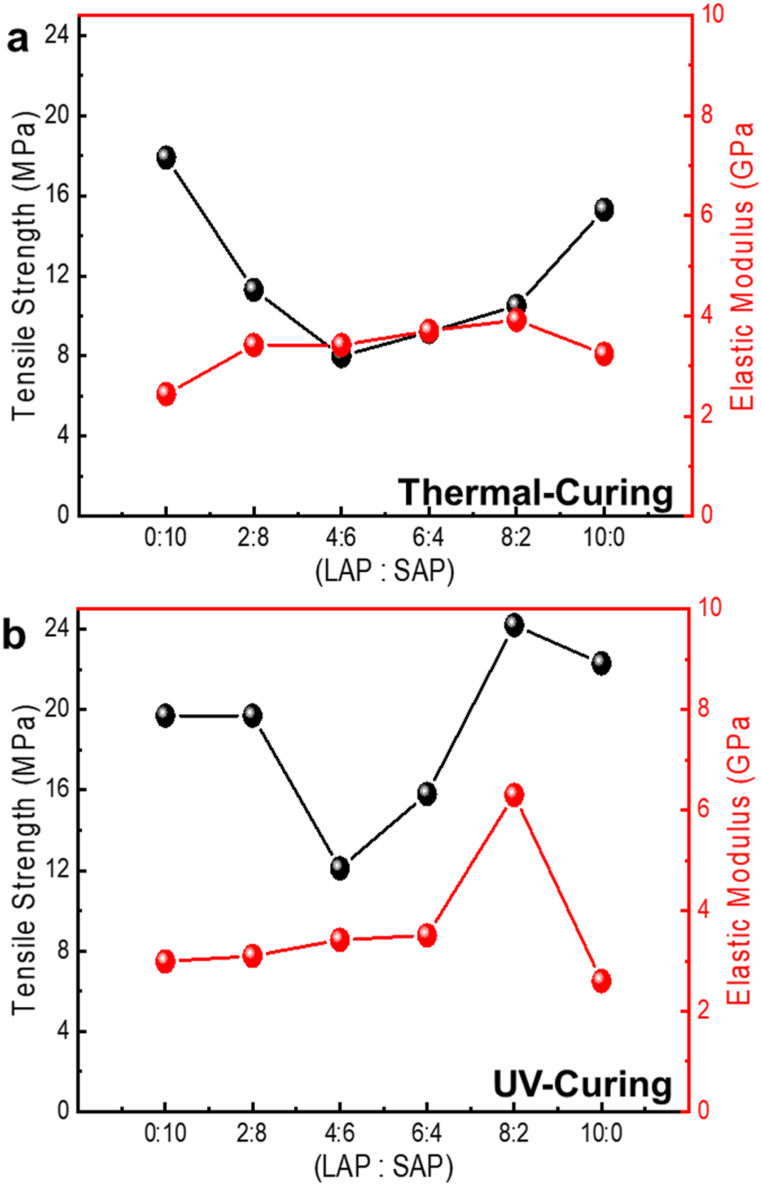
Tensile stress and elastic modulus values of a) thermal-cured and b) UV-cured AP-EPSH according to the ratios of LAP to SAP.

We conducted tensile tests for investigating the change in the mechanical properties of thermal- and UV-cured AP-EPSH with varying the ratio of LAP to SAP (Fig. 5 and S6). In thermal-cured AP-EPSH, with an increase in the concentration of LAP until 80% (LAP:SAP = 8:2), the tensile strength value (or facture strength) of AP-EPSH exhibited decreases (from 17.9 MPa to ∼10 MPa), while elastic modulus of AP-EPSH was gradually increased (from 2.44 GPa to 3.92 GPa) because of mechanical reinforcement by the increased packing density and effective reinforcement by small particles (i.e., SAPs) (Fig. 5a and Table 1) [32,33]. The tensile strength values increased when LAPs were further increased to 100% (LAP:SAP = 10:0) due to weakening mechanical reinforcement. This can be also confirmed by decreased elastic modulus to 3.24 GPa. In case of UV-cured AP-EPSH, tensile strength and elastic modulus were significantly increased (tensile strength: from 19.7 MPa to 24.2 MPa; elastic modulus: from 2.99 GPa to 6.30 GPa), which are much higher than those of thermal-cured composites because uniform distributed APs by UV-curing processes can make the effective mechanical reinforcement [16] (Fig. 5b). Changes of elongation with varying the ratios of LAP to SAP also exhibited similar trend, which are proof of mechanical reinforcement (Fig. S7). Therefore, we can confirm that UV-cured AP-EPSH with uniform distributed APs can induce effective mechanical reinforcement compared to thermal-cured AP-EPSH with large sedimented APs. Therefore, we conclude that the incorporation of APs with two different sizes enhances the tensile property of composites (i.e., tensile strength and elastic modulus) with the decreased elongation. Meanwhile, the UV-cured composites provide effective mechanical reinforcement (i.e., high elastic modulus) (Fig. S8).

**Table 1:** Mechanical properties values of thermal- or UV-cured AP-EPSH with varying the ratios of LAP to SAP.

Methods	LAP:SAP	Tensile Strength （MPa）	Elastic Modulus (GPa)
Thermal-Curing	0:10	17.9	2.44
	2:8	11.3	3.42
	4:6	8	3.42
	6:4	9.2	3.70
	8:2	10.5	3.92
	10:0	15.3	3.24
UV-Curing	0:10	19.7	2.99
	2:8	19.7	3.10
	4:6	12.1	3.42
	6:4	15.8	3.51
	8:2	24.2	6.30
	10:0	22.3	2.60

### 3.5 Electrical insulation properties of AP-EPSH

**Fig. 6:**
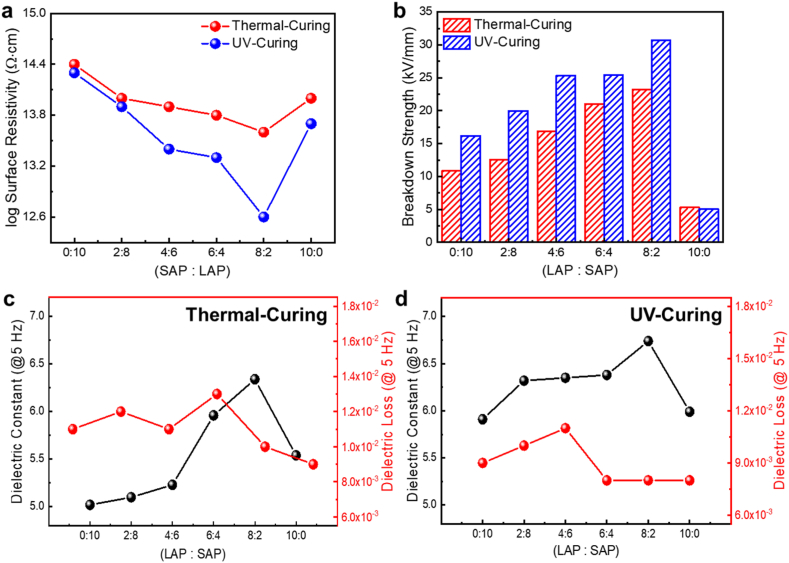
Electrical properties of thermal- and UV-cured AP-EPSH with varying the ratios of LAP to SAP. a) Surface resistivity, b) breakdown strength, and c-d) dielectric constant and dielectric loss.

Electrical insulation properties, such as electrical resistance, breakdown strength, and dielectric properties are crucial factors for using the composites as electronic packaging materials. Therefore, we investigated various insulation properties of thermal- and UV-cured AP-EPSH with varying ratios of LAP to SAP (Fig. 6). The electrical surface resistivity of thermal-cured AP-EPSH with 100% SAP was approximately 10^15^ Ω cm, which is relatively lower than that of the previously reported siloxane hybrid matrix and is attributed to the incorporation of AP with a high concentration in the matrix [5]. By increasing the portion of LAPs, the surface resistivity value (log scale) of AP-EPSH was continuously decreased (from 10^14^ Ω cm (logscale value: 14.4) to 10^13^ Ω cm (logscale value: 13.6) until the 80% incorporation of LAP (LAP:SAP = 8:2) caused by the increasing packing density in matrix (Fig. 6a). However, this value increased when only LAPs were added in siloxane hybrid matrix (100% of LAP), and it can be attributed to physical disconnection between LAPs with inserted SAPs, causing reduction of electrical conducting pathways. In case of UV-cured AP-EPSH, although similar behavior were able to be observed, all values were lower than those of thermal-cured AP-EPSH (from 10^14^ Ω cm (logscale value: 14.3) (LAP:SAP = 0:10) to 10^12^ Ω cm (logscale value: 12.6) (LAP:SAP = 8:2)), which is same reason in previous explained. The electrical breakdown strength values of thermal-cured AP-EPSH were increased continuously (from 10.88 kV/mm to 23.22 kV/mm) with the increasing portion of LAP until 80% incorporation of LAP (LAP:SAP = 8:2) (Fig. 6b). Alumina has a relatively low dielectric breakdown strength compared to that of siloxane-based hybrid matrix [34]. We can improve the breakdown strength of AP by combining it with the siloxane hybrid matrix. The breakdown strength of thermal-cured AP-EPSH was significantly decreased to 5.29 kV/mm, which is lower than that of AP-EPSH with 100% of SAP when LAPs were further added. This is due to dense particle accumulation by SAPs. For UV-cured AP-EPSH, the relatively higher breakdown strength values could be confirmed (from 16.2 kV/mm to 30.68 kV/mm); this indicate that the UV-cured siloxane-based matrix makes the effectively enhanced electrical breakdown strength of APs because of effective surrounding of APs with matrix. In addition, the stronger bond energy of the siloxane-based backbone in GPSH than that of the single carbon bond-based backbone in conventional polymers can be also effectively improved the breakdown strength [25,35]. Dielectric constant values of thermal-cured AP-EPSH were increased continuously (from 5.02 to 6.34) until 80% incorporation of LAP because of the high packing characteristics of composite with a high dielectric constant and the generation of continuous interconnection by SAPs (Fig. 6c). The dielectric loss property of thermal-cured AP-EPSH exhibited a similar trend to the dielectric constant results (from 0.009 to 0.013), which is attributed to the low dielectric loss property of alumina. The dielectric properties of AP-EPSH with 100% of SAP were similar to those of AP-EPSH with 100% of LAP because of the existence of high free volumes that can reduce the dielectric constant and relatively lower packing density than the other samples (AP-EPSH (LAP 100%): 5.02/0.009 and AP-EPSH (SAP 100%): 5.10/0.012) [33]. Although a similar tendency of UV-cured AP-EPSH was also shown in which increased dielectric constant until 80% (from 5.91 to 6.14) and re-decreased after 100% of LAP was added, the effect by APs was higher than that of thermal-cured AP-EPSH (Fig. 6d); higher dielectric constant and lower dielectric loss. This indicates uniform distribution and compact surrounding of APs by matrix, which can lead to increase dielectric constant and decrease dielectric loss (Fig. S9).

By combining various experimental results and packing theory, we concluded that reinforcement with APs of two different sizes increase packing density (especially, at a LAP-to-SAP ratio of 8:2); this greatly enhances the mechanical and thermal properties. However, these induce low surface resistivity and high dielectric constant because of the electrical properties of APs. Furthermore, we found that UV-curing process makes uniform distribution of APs and effective reinforcement compared to thermal-curing process, which significantly improved various properties. Our approach for fabricating particle-based composites has a great potential for future application to electronics packaging and various insulation materials that require high thermal conductivity and mechanical robustness.

## 4 Conclusion

In conclusion, we developed AP-incorporated cycloaliphatic epoxy-phenyl-based siloxane hybrid composite (AP-EPSH) to obtain high thermal, mechanical, and insulation properties. The sol-gel derived siloxane hybrid matrix with highly condensed robust inorganic networks was employed. To maximize the packing density of AP-EPSH, bimodal sized APs with high thermal conductivity were introduced. We investigated the effect on the various physical properties of AP-EPSH according to curing mechanism (thermal- or UV-curing) and varying the ratio of LAP and SAP. The greatly improved thermal conductivity with high thermal stability, enhanced mechanical robustness (i.e., increased elastic modulus and decreased elongation) by incorporating bimodal sized APs in the siloxane hybrid matrix were obtained, while increased the dielectric properties (dielectric constant and dielectric loss) and decreased the surface resistivity were able to be confirmed. The experimental results indicated that all property changes were maximized when the ratio of LAP to SAP was 8:2 because of the packing density of AP-EPSH was the highest among the other ratios. This can be explained via calculations using the packing theory of composite with two different size filler particles. Furthermore, UV-curing process exhibited more effective than thermal-curing process, which is induced by uniform distribution without sedimented and aggregation APs. Following these, we obtained superior thermal conductivity (>11 W/m K), high mechanical strength (tensile strength = 24.2 MPa, elastic modulus = 6.30 GPa), electrical breakdown strength (30.68 kV/mm), and enhanced thermal stability (T_d5wt%_ = 380 °C, with 90% residual weight after test and CTE = 20.23 ppm/K) by using UV-curing process. Therefore, we achieved the trade-off characteristics of high thermal conductivity with high electrical insulation properties by optimizing composition of composites with high packing density. We expect that our approach for fabricating AP-based composite has a significant potential toward high-performance electrical packaging and insulation materials that require high thermal conductivity and thermal/mechanical robustness.

## CRediT authorship contribution statement

**Chan Soo Kim:** Data curation, Formal analysis, Investigation, Validation. **Junho Jang:** Conceptualization, Data curation, Formal analysis, Investigation, Validation, Visualization, Writing – original draft, Writing – review & editing. **Hyeon-Gyun Im:** Formal analysis, Investigation, Validation, Visualization, Writing – review & editing. **Seogyoung Yoon:** Funding acquisition, Project administration, Supervision, Validation, Writing – review & editing. **Dong Jun Kang:** Conceptualization, Formal analysis, Funding acquisition, Project administration, Supervision, Writing – review & editing.

## Declaration of competing interest

The authors declare that they have no known competing financial interests or personal relationships that could have appeared to influence the work reported in this paper.
