# Supplementary material for: Preparation and performance of alumina/epoxy-siloxane composites: A comparative study on thermal- and photo-curing process
Source: Heliyon. 2024 Mar 4;10(5):e27580. doi: 10.1016/j.heliyon.2024.e27580 (PMC10940936; doi:10.1016/j.heliyon.2024.e27580)
Supplement: Multimedia component 1 [file mmc1.docx]

Supplementary Material

**Preparation and Performance of Alumina/Epoxy-Siloxane Composites: A Comparative Study on Thermal- and Photo-Curing Process**

*Chan Soo Kim,^1,3†^, Junho Jang^2†^, Hyeon-Gyun Im^1^, Seogyoung Yoon^3*^, Dong Jun Kang^1*^*

^1^Insulation Materials Research Center, Electrical Materials Research Division, Korea Electrotechnology Research Institute (KERI), Changwon-si 51543, Republic of Korea

^2^Wearable Materials Technology Center (WMC), Department of Materials Science and Engineering, Korea Advanced Institute of Science and Technology (KAIST), Daejeon 34141, Republic of Korea

^3^School of Materials Science and Engineering, Pusan National University, Busan 46241, Republic of Korea

^†^These authors contributed equally to this work

^*^Corresponding author: Dong Jun Kang (kangdj@keri.re.kr) and Seogyoung Yoon (syy3@pusan.ac.kr)


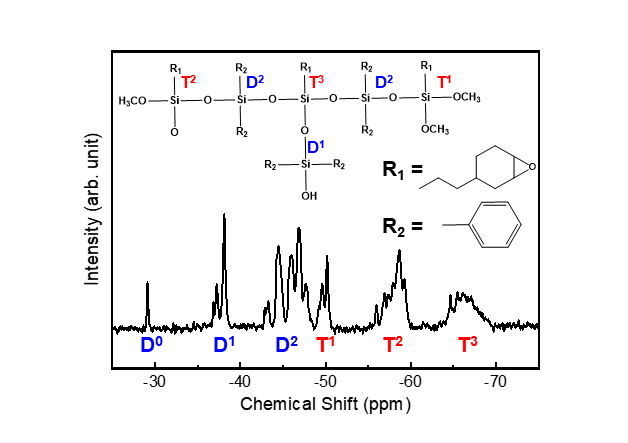


Fig. S1. ^29^Si-NMR analysis of EPSR


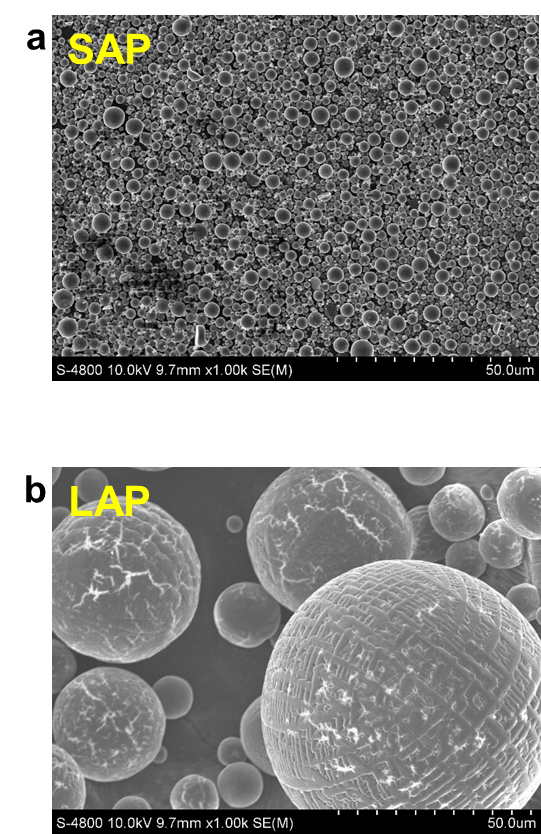


Fig. S2. SEM images of a) SAP and b) LAP.


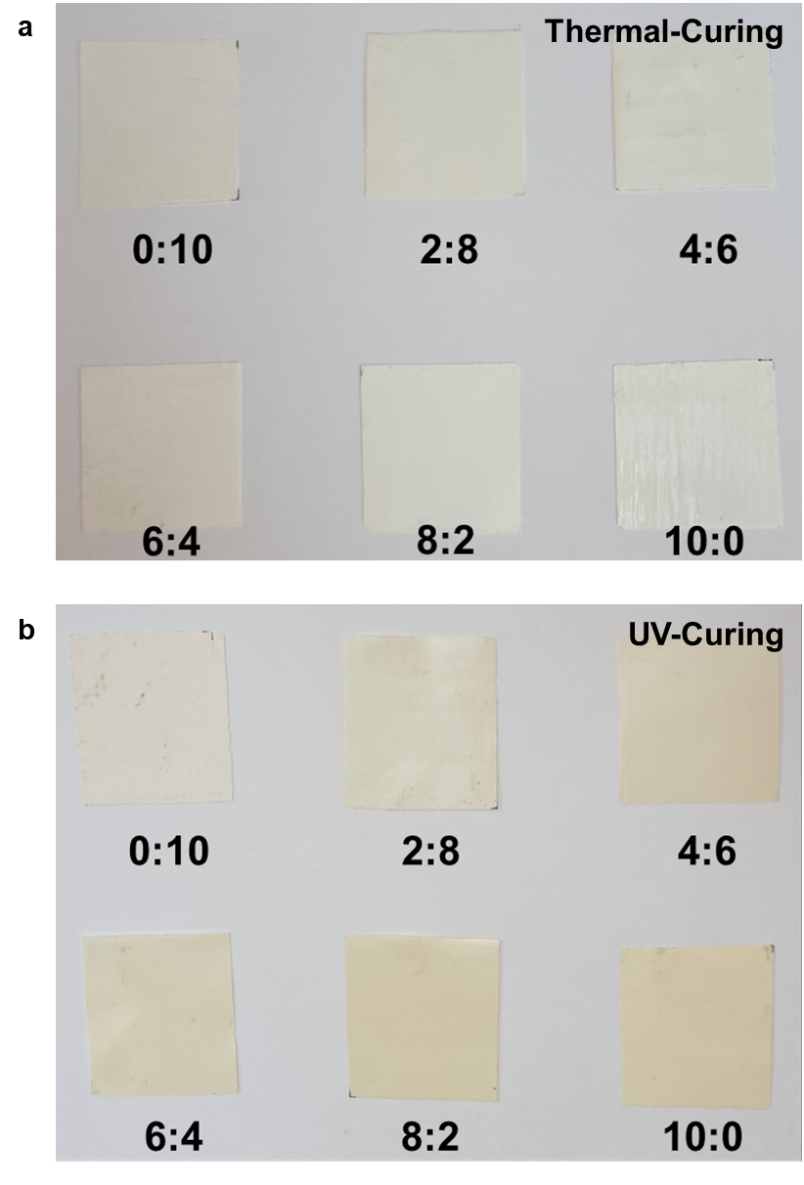


Fig. S3. Photographs of a) thermal- and b) UV-cured AP-EPSH according to changing the ratio of LAP to SAP.


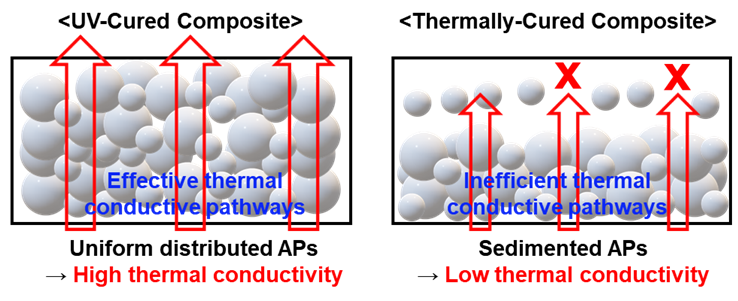


Fig. S4. Comparison of thermal conduction mechanism of thermal- and UV-cured AP-EPSH.


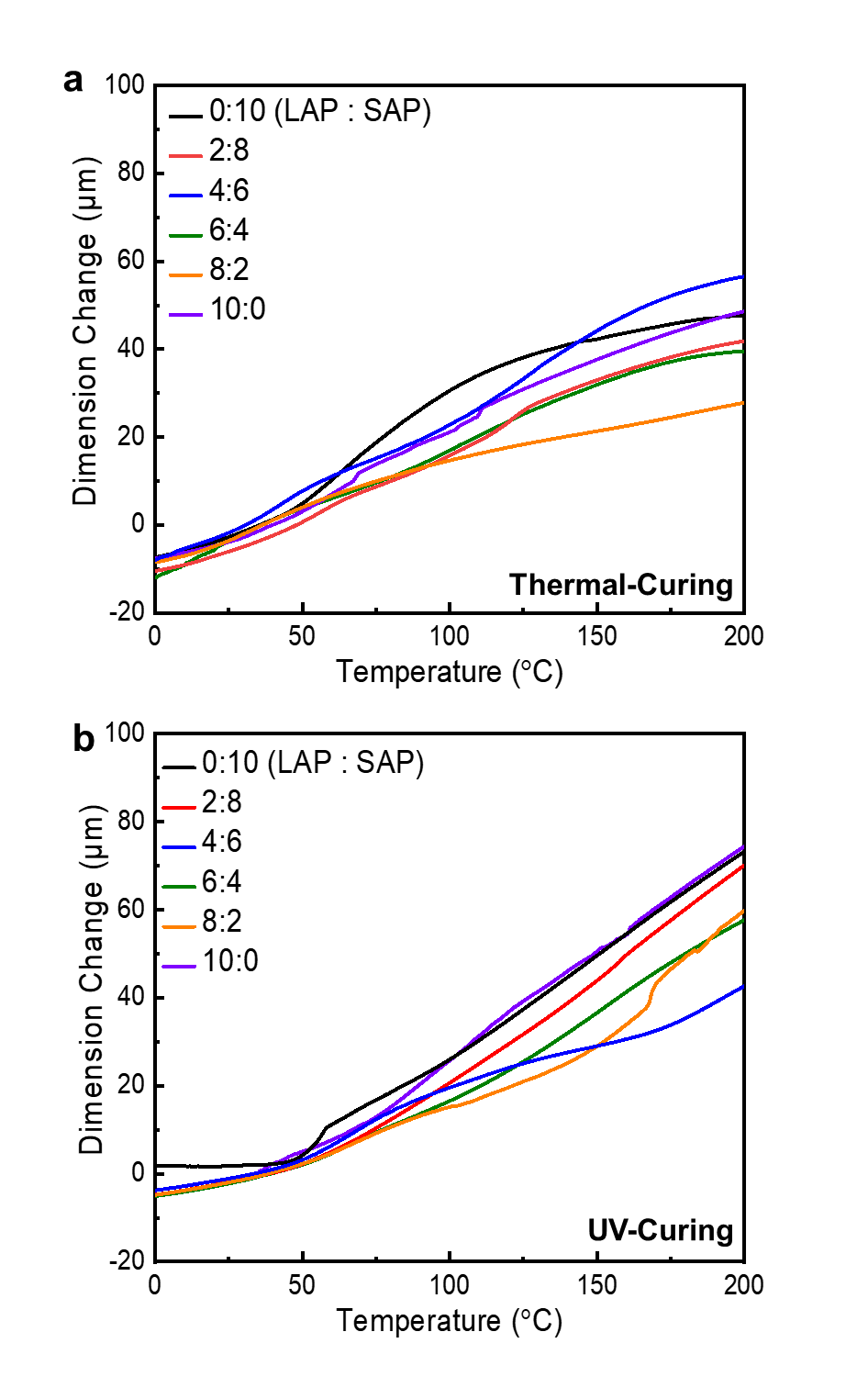


Fig. S5. TMA analysis graph of a) thermal- and b) UV-cured AP-EPSH with varying the ratio of LAP to SAP.


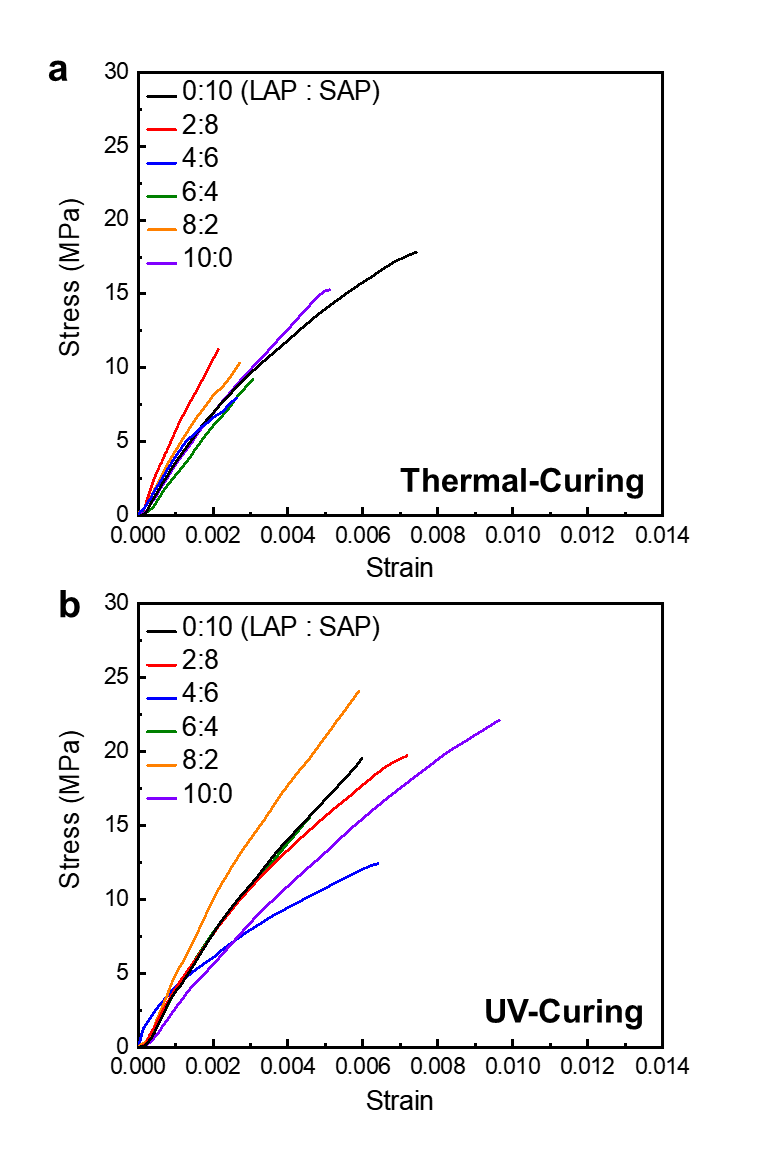


Fig. S6. Tensile stress–strain curve of a) thermal- and b) UV-cured AP-EPSH with varying the ratios of LAP to SAP.

Fig. S7. Change of elongation of thermal- and UV-cured AP-EPSH with varying the ratios of LAP to SAP.

Fig. S8. Schematic illustration of mechanical reinforcement by combining EPSH (softer) and AP fillers (harder).

Fig. S9. Schematic illustration of mechanical of changing dielectric properties according to the ratio of LAP to SAP and different curing processes.

**Table S1.** 5% weight loss temperature and residual weight of thermal- and UV-cured AP-EPSH with varying the ratio of LAP to SAP.

| **Methods** | **Ratio of LAP to SAP** | **T_d5%_** | **Residual Weight** |
| --- | --- | --- | --- |
| Thermal-Curing | 0:10 | 378.59 | 90.40 |
|  | 2:8 | 391.93 | 90.29 |
|  | 4:6 | 393.58 | 90.40 |
|  | 6:4 | 383.96 | 90.02 |
|  | 8:2 | 377.27 | 89.95 |
|  | 10:0 | 387.23 | 89.36 |
| UV-Curing | 0:10 | 367.09 | 89.51 |
|  | 2:8 | 373.54 | 89.69 |
|  | 4:6 | 368.39 | 89.26 |
|  | 6:4 | 380.51 | 90.15 |
|  | 8:2 | 377.93 | 89.62 |
|  | 10:0 | 384.38 | 2.60 |
